# Host Immune Responses in HIV-1 Infection: The Emerging Pathogenic Role of Siglecs and Their Clinical Correlates

**DOI:** 10.3389/fimmu.2017.00314

**Published:** 2017-03-23

**Authors:** Joanna Mikulak, Clara Di Vito, Elisa Zaghi, Domenico Mavilio

**Affiliations:** ^1^Unit of Clinical and Experimental Immunology, Humanitas Clinical and Research Center, Rozzano, Italy; ^2^Istituto di Ricerca Genetica e Biomedica, UOS di Milano, Consiglio Nazionale delle Ricerche (UOS/IRGB/CNR), Rozzano, Italy; ^3^Department of Medical Biotechnologies and Translational Medicine (BioMeTra), University of Milan, Milan, Italy

**Keywords:** host-pathogen interactions, NK cells, monocytes/macrophages, dendritic cells

## Abstract

A better understanding of the mechanisms employed by HIV-1 to escape immune responses still represents one of the major tasks required for the development of novel therapeutic approaches targeting a disease still lacking a definitive cure. Host innate immune responses against HIV-1 are key in the early phases of the infection as they could prevent the development and the establishment of two hallmarks of the infection: chronic inflammation and viral reservoirs. Sialic acid-binding immunoglobulin-like lectins (Siglecs) belong to a family of transmembrane proteins able to dampen host immune responses and set appropriate immune activation thresholds upon ligation with their natural ligands, the sialylated carbohydrates. This immune-modulatory function is also targeted by many pathogens that have evolved to express sialic acids on their surface in order to escape host immune responses. HIV-1 envelope glycoprotein 120 (gp120) is extensively covered by carbohydrates playing active roles in life cycle of the virus. Indeed, besides forming a protecting shield from antibody recognition, this coat of N-linked glycans interferes with the folding of viral glycoproteins and enhances virus infectivity. In particular, the sialic acid residues present on gp120 can bind Siglec-7 on natural killer and monocytes/macrophages and Siglec-1 on monocytes/macrophages and dendritic cells. The interactions between these two members of the Siglec family and the sialylated glycans present on HIV-1 envelope either induce or increase HIV-1 entry in conventional and unconventional target cells, thus contributing to viral dissemination and disease progression. In this review, we address the impact of Siglecs in the pathogenesis of HIV-1 infection and discuss how they could be employed as clinic and therapeutic targets.

## Introduction

Immunoglobulin-like receptors constitutively present on cellular surface play a key role in the immune recognition of both cell targets and pathogens due to their high plasticity that makes them easily adaptable to adopt an infinite range of molecular structures. In this regard, glycans expressed by several microbes are recognized by several immunoglobulin type-I lectins, including sialic acid-binding immunoglobulin-like lectins (Siglecs). Siglecs belong to a family of transmembrane proteins that display a variable numbers of C2-set immunoglobulin domains (from 1 in CD33 to 16 in Siglec-1), an amino-terminal V-set immunoglobulin domain that recognizes sialic acid residues (1–3) and a conserved arginine residue that binds carbohydrates. Fifteen different functional Siglecs have been identified in humans, including Siglec-12 that has lost the ability to bind sialic acids. They can be classified in two main subgroups: the first one comprises the evolutionary conserved Siglecs-1, -2, -4, and -15 that differ from the second cluster of rapidly evolving Siglecs. This latter type-I lectin subgroup includes Siglec-3, -5 to -11, -14, and -16 sharing structural similarities with the first identified CD33/Siglec-3 (4–6). Siglecs are constitutively expressed mostly on cells derived from the hematopoietic lineage and belonging to innate immunity, such as monocytes/macrophages, neutrophils, basophils, eosinophils, dendritic cells (DCs), and natural killer (NK) cells (5). In contrast, adaptive immune cells such as T lymphocytes have very low or undetectable surface levels of these type-I lectins (7). Of note, the expression of distinct member of Siglec family can be either restricted to specific immune cells (i.e., Siglec-1 and -11 constitutively present only on macrophages) (5, 8, 9) or shared by several myeloid and lymphoid populations (i.e., Siglec-7 and -9 expressed on NK cells, monocytes and macrophages or Siglec-5 on monocytes, B cells, and neutrophils) (5, 10–12).

In regard to Siglec binding to their putative ligands, each one of these lectin-type molecules has a preferential specificity for sialic acid residues, and the recognition depends on both glycan structures and glycan modifications associated with sulfation, acetylation, and glycosylation reactions (13, 14). Sialic acids comprise a family of more than 50 carbohydrates that share a nine-carbon backbone (C1-9) representing the terminal unit of glycoproteins and glycolipids (gangliosides) phylogenetic conserved among different species including humans (15). Siglec-binding sites are usually masked at the cellular surface due to *cis* interactions with highly expressed low affinity sialic acids. Their unmasking in response to cellular activation or sialidase treatment cleaves the *cis* low affinity ligands and allows free interactions in *trans* with highly glycosylated ligands. Nonetheless, *trans* interactions can also occur when Siglecs encounter other cells or pathogens expressing higher affinity ligands competing with *cis* (16, 17). Following the binding with these sugars, Siglecs regulate and control inflammatory responses by setting appropriate thresholds of immune activation (5, 18–20). Indeed, Siglec cytosolic tails mostly include immune-receptor tyrosine-based inhibitory motifs (ITIMs) or ITIM-like domains, whose engagement inhibits leukocyte proliferation, induces apoptosis, modulates endocytosis and the production of inflammatory cytokines.

In the context of host–pathogen interactions, sialic acids and their glycan residues expressed on the envelopes of several pathogens serve as recognition molecules interacting with lectin-type molecules such as Siglecs. This is one of the established mechanisms of viral entry in immune cells, as demonstrate with highly pathogenic A, B, and C strains of influenza that encode hemagglutinin enriched with sialic acid residues (21, 22). The engagement of Siglecs in response to their recognition of microbial glycan residues has been first described as a mechanism to dampen host immune responses following the encounter with inflammatory stimuli (23–26). Later on, it became evident that pathogens took advantage of the Siglec recognition by evolving and expressing sialic acids on their surface in order to escape host inflammatory responses (20, 27). More recently, a novel and emerging working hypothesis postulates that CD33-related Siglecs also evolved in response to pathogen manipulation by associating their intracellular domains with either immune-receptor tyrosine-based activation motifs (ITAM) or unconventional activating from of ITIMs in Siglec-7 (17, 20, 28–30).

Several lines of evidence indicate that also HIV-1 employs sialic acid associated-mechanisms to facilitate its viral entry in target cells. Indeed, HIV-1 envelope (env) is equipped with an N-terminal glycoprotein 120 (gp120) heavily conjugated with sialic acids residues that, besides forming a protecting shield from antibody (Ab) recognition, also enhance virus infectivity and dissemination by binding sugar-binding receptors expressed on host immune cells (31–39). Indeed, several human lectin-type receptors including galectins (40, 41), defensins (42–44), and others (45–51) have been shown to bind HIV-1 env by recognizing glycans expressed on gp120. The identification and characterization of these sialic acid-binding receptors also explain the different susceptibility to HIV-1 to infect cells expressing different repertoires of Siglecs (52). Although these lectin-type receptors were identified in the early 1980s, only recently their interactions with HIV-1 have been disclosed. In particular, Siglec-1 (53–55) and Siglec-7 (52, 56) have been reported to bind HIV-1 envelope, thus inducing and/or facilitating HIV-1 infection and disease progression. This review provides an updated summary in regard the pathogenic role of Siglecs and also discusses how they could be employed as clinic and therapeutic targets.

## Kinetics of Siglecs During the Course of HIV-1 Infection

Human NK cells are known as effector-cells capable of killing potentially dangerous non-self targets (i.e., viral-infected, tumor-transformed, or allogeneic cells) and produce pro-inflammatory cytokines such as interferon-γ (IFN-γ) upon activation and in the absence of a prior sensitization to specific antigens. NK cell recognition of autologous cell targets is mediated by a large family of inhibitory NK cell receptors (iNKRs) including killer cell immunoglobulin-like receptors and C-type lectins that recognize different alleles of major histocompatibility complex of class I (MHC-I) (i.e., missing-self hypothesis). Decreased expression or absence of self-MHC-I on viral-infected or tumor-transformed or allogeneic cells trigger NK cell effector functions *via* the engagement of large family of activating NKRs (aNKRs) that bind their putative ligands expressed on cellular targets (57–59). Even though the inhibitory signals are considered dominant over the activating ones, the dynamic balance between iNKRs and aNKRs is key for the regulation of NK-cell-mediated clearance of viruses and tumors (60–62). NK cells are CD3^neg^/CD19^neg^/CD14^neg^ lymphocytes whose phenotype is defined by the expression of CD56 and CD16 (56, 59). In healthy subjects, the largest subset of circulating NK cells (up to 90%) is CD56^dim^/CD16^pos^, has a high cytolytic potential, and can also produce high levels of pro-inflammatory and antiviral cytokines upon stimulation. The second circulating subset of immune-regulatory CD56^bright^/CD16^dim-neg^ NK cells (up to 10–15%) is able to secrete high amount of pro-inflammatory cytokines while displaying poor cytotoxicity (59, 63, 64).

The first studies assessing the frequencies and the functions of NK cells in HIV-1 infected patients with high levels of ongoing viral replication reported an overall decrease of both their percentage and absolute number in the peripheral blood. Either apoptosis or cellular migration to peripheral tissues has been proposed as possible mechanisms driving the depletion of circulating NK cells during the course of HIV-1 infection (65, 66). Only later, it became evident that HIV-1 viremia does not reduce the absolute number of circulating NK cells but rather induce a pathologic redistribution of their subsets since from the early stages of the infection. Indeed, others and we identified and characterized an anergic population of CD56^neg^/CD16^pos^ (CD56^neg^) NK cells expanded in viremic patients and counterbalancing the reduction of the CD56^dim^/CD16^pos^ cytolytic NK cell subset. An early depletion of the immune-regulatory CD56^bright^/CD16^neg^ NK cells has been also described (64, 67–69). This subset of CD56^neg^ NK cells in HIV-1 infection is characterized by an imbalanced repertoire of NKRs associated with a remarkably impaired cytotoxic activity and secretion of antiviral cytokines/chemokines (68, 70). These pathological features explain, at least in part, the impairments of CD56^neg^ NK cells in the clearance of both tumor-transformed and viral-infected cell targets as well as their defective interactions with autologous DCs (71–73). The mechanisms driving the expansion of this pathologic CD56^neg^ NK cells during the course of HIV-1 infection remain unknown, although several lines of evidence indicate that this phenomenon is not HIV-1 specific. Indeed, high frequencies of CD56^neg^ NK cells have been reported in other viral infections [such as human cytomegalovirus or hepatitis C virus (HCV)] and autoimmune diseases (such as myasthenia gravis or dermatomyositis) (64). Hence, it is conceivable to hypothesize that persistent inflammation characterizing several human chronic immunological diseases might be one of the factors inducing the expansion of CD56^neg^ NK cells.

The down-modulation of CD56 is not the only NK surface marker whose surface level is altered by high levels of ongoing HIV-1 replication. In fact, also the expressions as well as the functional relevance of several aNKRS and iNKRs are affected by HIV-1 viremia (62, 74). Indeed, these markedly dysfunctional CD56^neg^ NK cell subsets express high levels of iNKRS and low levels of aNKRs, a phenomenon that contributes, at least in part, to a defective control of HIV-1 replication and to disease progression (64, 72). The HIV-1-induced pathologic modulation of CD56 and NKR surface expression does not occur during the acute phase of the infection but require a chronic exposure of NK cells to chronic viral replication (75). In contrast, the down-modulation of Siglec-7, a iNKRs constitutively expressed on the majority of NK cells, is detectable since from the earliest phases of HIV-1 infection and precedes the phenotypic changes of CD56 and NKRs on NK cells (56). This phenomenon makes it possible to identify two distinct subsets present in different stages of HIV-1 infection: the Siglec-7^neg^/CD56^dim^ NK population only present in the early phases of HIV-1 infection and the Siglec-7^neg^/CD56^neg^ NK cell subset that become detectable several months later when the disease enters in its chronic phase. All these HIV-1-induced phenotypic changes are reversible following the administration of a successful antiretroviral therapy (ART) and the restoration of Siglec-7 expression on NK cells is much faster compared to that of CD56, aNKRs, and iNKRs. The impact of viral replication in inducing these phenotypic abnormalities is also confirmed by the experimental evidence showing that NK cells from those HIV-1-infected patients with a low/undetectable levels of viral replication that do not progress to AIDS (i.e., long-term non-progressors or LTNP) are similar and undistinguishable from the ones of uninfected healthy individuals (56). Although Siglec-7 is an iNKRs containing ITIM-like domains in its cytosolic tail, it has been also reported that Siglec-7^pos^ NK cells are more functionally relevant compared to their Siglec-7^neg^ counterparts. Indeed, the expression of Siglec-7 in NK cells is associated with higher levels of aNKRs (i.e., CD16, NKp30, NKp46) and lower levels of iNKRs (i.e., CD94/NKG2A, CD158b) (76). This likely explains the fact that NK cells still retain a certain degree of antiviral and antitumor functions in the acute phases of HIV-1 infection, while become anergic and highly dysfunctional only in the chronic stages of the disease featuring the expansion of the Siglec-7^neg^/CD56^neg^ NK cells (56).

A soluble form of Siglec-7 (sSiglec-7) resulted to be increased in the sera of HIV-1-infected patients and could serve as an additional clinical biomarker to monitor disease progression. In fact, higher serum levels of sSiglec-7 directly correlate with HIV-1 viremia and parallel the expansion of Siglec-7^neg^ NK cells in HIV-1-infected patients, thus suggesting that the continuous exposure to ongoing viral replication induce the shedding of Siglec-7 receptor from NK cell surface (52, 56). Among circulating immune cells constitutively expressing this C-lectin-type molecule (i.e., NK cells and monocytes) (5), the main source of sSiglec-7 seems to be NK cells because a subset of Siglec-7^neg^ monocytes has never been reported during the course of HIV-1 infection (52). Similarly, it has been recently shown that also the stimulation *in vitro* of NK cells with HCV induces both a significant release of sSiglec-7 in culture supernatant and a decrease of Siglec-7 expression from NK cell surface (77). Further investigations are required to identify the cellular and molecular mechanisms that certain viruses employ to induce a shedding of this C lectin-type receptor selectively on NK cells during the acute phases of the infection.

Siglec-1 is a sialoadhesin (i.e., an adhesion molecule present on cell surface and binding sialic acid residues) constitutively expressed on monocytes and macrophages whose expression is remarkably increased on HIV-1 infected monocytes (54, 78, 79). These higher amount of Siglec-1 on CD14^pos^ cells are induced by IFN-α and IFN-γ, occur rapidly after infection, enhance their susceptibility to infection, and are maintained during the following chronic phases of HIV-1 disease. In particular, the expression of Siglec-1 on monocytes appears to be higher in AIDS patients compared to that of asymptomatic HIV-1-infected individuals and correlates with plasma viral load. These findings suggest that high frequencies of Siglec-1^pos^/CD14^pos^ monocytes are associated with disease progression (80). In addition, higher surface levels of Siglec-1 on monocytes correlate with immune activation as the treatment of these cells with IFNs or bacterial compounds such as lipopolysaccharide increases the surface amount of this type-I lectin. This is consistent with the condition of chronic inflammation and immune activation induced by the presence of HIV-1 viremia that also triggers the expansion of a subset of CD14^pos^/CD16^pos^-activated monocytes (54, 78, 81–84). Hence, although having different kinetics during the course of HIV-1 infection, both the expressions of Siglec-1 and Siglec-7 are greatly influenced by viral replication and can be used as clinical tools to identify and monitor acute and chronic clinical stages of the infection.

## Siglecs in the Pathogenesis of HIV-1 Infection

In addition to CD4^pos^ T lymphocytes that represent the main cell target of HIV-1 infection, the virus is able to also infect immune cells of myeloid origin such as DCs and tissue-resident macrophages (71, 85). These two latter antigen-presenting cells express both CD4 and the chemokine co-receptors CCR5 and CXCR4, which are required for a productive HIV-1 infection (86). In particular, different Siglecs have been reported to play key roles in both binding the virus and facilitating its viral entry in monocytes and macrophages due to the fact that HIV-1 env-gp120 is extensively covered by carbohydrates first processed in the endoplasmic reticulum and then further modified in the *trans*-Golgi apparatus (31–35). The high degree of sialylation on HIV-1 envelope, other than representing a protecting shield that hamper Ab recognition of viral antigens, is also highly permissive and facilitates HIV-1 infection of target cells (33, 87–89). Indeed, given the high avidity and affinity of Siglecs for sialic acid residues, the viral env-gp120 (in particular within the V1/V2 domain) represents an important Siglec-binding site (90). Different from CD4^pos^ T cells though, monocytes and macrophage are resistant to HIV-1-mediated cytopathic effects as there is no depletion of these cells either in peripheral blood or tissues of HIV-1-infected patients. Moreover, macrophage can serve as cellular storage of accumulating virions in their sub-cellular regions that are termed “virion containing compartment” and whose origin is still unclear (91, 92). The identification of VVCs gave rise among the scientific community to the “Trojan horse” metaphor meaning that the virus can be hidden in infected macrophages that, in turn, can disseminate infectious particles when interplaying with CD4^pos^ T cells and/or in response to environmental signals (i.e., cytokines or damage-associated molecular patterns). Several experimental evidence confirmed the existence of this “Trojan horse” in *in vitro* infected monocytes/macrophages, in the brain of AIDS patients with HIV encephalitis (93, 94) as well as in macaques infected with Simian immunodeficiency virus (SIV) (95, 96). It is still being debated whether HIV-1 infection of macrophages leads to the establishment of an additional reservoirs of latently infected cells contributing to boost viral replication and disease progression in patients undergoing ART interruption (97). In supports to this hypothesis, it has been reported that the functional polarization of monocyte-derived macrophages (MDM) into M1 cells upon exposure to pro-inflammatory signals (i.e., IFN-γ and TNF-α) leads to a state of quasi-latency upon HIV-1 infection (98). Moreover, recent evidence demonstrates that M2 macrophages are more permissive to HIV-infection/replication compared to their M1 counterparts. This is associated with the fact that the stimulation with macrophage colony-stimulating factor induces both *in vitro* and *in vivo* the expression on macrophage of several cellular receptors required for viral entry, including CD4, CCR5, and Siglec-1 (90, 99).

**Figure 1:**
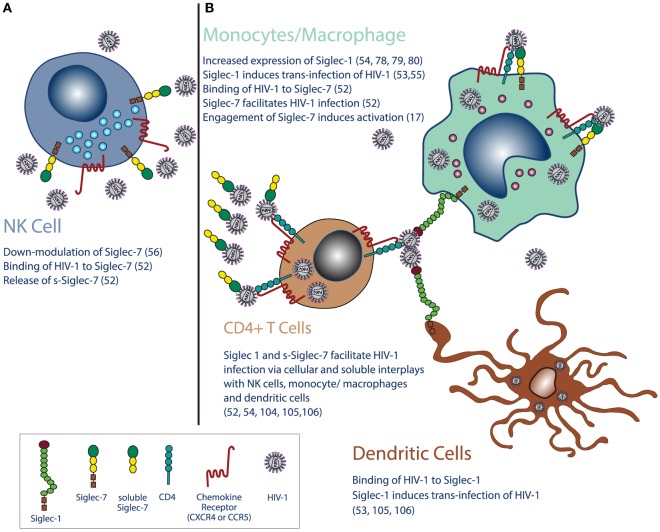
**Siglecs in the pathogenesis and clinical outcomes of HIV-1 infection**. **(A)** Chronic HIV-1 infection leads to the downregulation of Siglec-7 expression on natural killer (NK) cells and increases plasma levels of its soluble form of Siglec-7 (sSiglec-7). **(B)** Moreover, HIV-1 monocytes/macrophages induces higher expressions of Siglec-1, CD4, and chemokine co-receptors (CCR5 and CXCR4) on monocytes and macrophages, while do not affects the constitutive high surface levels of Siglec-7 on the same cells. Siglec-1 is also expressed on mature dendritic cells (mDCs), monocytes, and macrophages, and high levels of HIV-1 viremia increase even more its surface expression on the latter two cell types. Due to their distribution on immune cells and to their ability to bind the viral envelope glycoprotein 120 heavily conjugated with sialic acids residues, Siglec-7 and Siglec-1 are highly relevant in the pathogenesis of HIV-1 infection. Indeed, after the binding of HIV-1 to Siglec-1, both mDCs and monocytes/macrophages retain virions with different mechanisms, a phenomenon enhancing the HIV-1 infection of CD4^pos^ lymphocytes. Moreover, the HIV-1 uptake by Siglec-1 and Siglec-7 further contribute to the spreading of infection by using both DCs and monocyte/macrophages as cellular-infected carriers of the virus. The shedding of Siglec-7 from NK cells in the presence of high levels of HIV-1 viremia also significantly increases HIV-1 viral entry in Siglec-7^neg^/CD4^pos^ T cells. Finally, the engagement of Siglec-7 on monocytes and macrophages promotes cellular activation in monocytes and macrophages and contributes to the establishment of persistent inflammation in HIV-1-infected patients. These abnormalities in Siglec phenotype and functions in HIV-1 infection can be used as clinical tools to monitor disease progression or can be targeted to develop innovative therapeutic approaches to limit viral spreading and disease progression.

The first evidence of a direct contribution of Siglecs to HIV-1 pathogenesis has been reported in monocytes with the Siglec-1-mediated uptake of HIV-1 in a sialic acid-dependent mechanism (54). Indeed, Siglec-1 can bind HIV-1 and promote a *trans*-infection of monocytes. Unlike the other Siglecs in which the sialic acid binding sites could be masked by ligands expressed in *cis*, Siglec-1 is equipped with a larger extracellular domain that allow both cell-to-cell and cell-to-virus interactions. The discovery of this mechanism made it possible to understand that Siglec-1 is able to directly interact with HIV-1 and contributes to the spreading of HIV-1 infection (5). Moreover, Siglec-1 on macrophages can also function as an adhesion molecule carrying sialic acid conjugates to T cells (100, 101). Hence, the main current working hypothesis postulates that monocytes and macrophages interplay at the same time with viral envelope and target cells *via* Siglec-1 that bridges HIV-1 in close proximity to CD4 and chemokine co-receptors (Figure 1). This interaction would overcome the electrostatic repulsive forces created by the negative charges present on both virus envelope and cell surface, thereby unmasking the receptors involved in the internalization of HIV-1 and promoting viral entry. In support of this hypothesis, several studies showed that removal of sialic acids from cell surface or viral envelope with neuraminidase treatment increases the susceptibility of target cells to be infected and form cellular syncytia (33, 39, 102, 103). Moreover, a recent report demonstrated in an *in vivo* rodent model of HIV infection that the virus can disseminate over long distance in a cell-free manner in the blood and lymph and can be captured by Siglec-1^pos^ macrophages mainly located at the interface between lymphoid tissues and fluids, thus allowing and facilitating the trans-infection of lymphocytes (104).

Siglec-1 also mediates HIV-1 *trans*-infection of mature DCs (mDCs) and contributes to viral dissemination *via* the establishment of immunological synapses between mDCs and T cells in the early steps of the infection at mucosal sites (53). In this regard, intra-vaginal inoculation of SIV recruit plasmacytoid DCs to lymphoid tissue and increases tissue levels of IFN-α. In turn, this pro-inflammatory cytokine induces the maturation of DCs and their ability to mediate HIV-1 *trans*-infection of CD4^pos^ T cells and of themselves *via* Siglec-1 (105, 106). The interactions between Siglec-1 expressed on mDCs and sialic acids residues present on viral membrane or exosomes also represents a host immune mechanism employed by mDCs to process and present antigens to CD4^pos^ T cells. HIV-1 evolved to take advantage of this physiologic pathway and used mDCs as a carrier of infection (53). Recently, it has been proposed that HIV-1 particles do not only parasite mDCs in their cellular compartments, but virions are also retained on surface-connected plasma membrane invaginations (107) (Figure 1).

Monocyte-derived macrophages express different Siglecs that, other than binding HIV-1, have been also reported to enhance the susceptibility of MDMs to be infected by the same virus (52, 55). Siglec-1 leads this process by serving as the most effective MDM surface receptor up-taking HIV-1 envelope, even though its degree of expression on these cells is significantly lower compared to that of Siglec-3 and -9. As previously mentioned, this is likely due to the fact that Siglec-1 has a larger size with 17 immunoglobulin domains size and is present in an un-masked state, while Siglec-3 and -9 are masked on MDM cell surface and display a great affinity toward gp120 only after neuraminidase treatment (55). It has been recently reported that also Siglec-7 acts as a membrane-bound molecule that contributes to enhance HIV-1 infection of MDMs (52). Several studies showed that both monocytes and macrophages constitutively express high levels of Siglec-7 (5, 10, 20, 24, 25, 52), while only one report claiming a very low expression of this type-I molecule on MDMs (53). Similar to Siglec-1, Siglec-7 binds gp120 present on viral envelope and increases the viral entry of HIV-1 in MDMs. Indeed, the masking of Siglec-7 greatly reduce the amount of HIV-RNA in MDMs, thus decreasing their susceptibility to infection (52) (Figure 1).

Siglec-7 has been recently reported of being able to increase the susceptibility to HIV-1 infection even its soluble form (52). Indeed, Siglec-7-Fc fusion protein significantly increases HIV-1 viral entry in Siglec-7^neg^/CD4^pos^ T cells. This phenomenon is highly relevant in the course of active and chronic HIV-1 infection since AIDS patients show increased serum levels of sSiglec-7. Again, although a direct experimental evidence is still lacking, it has been hypothesized that sSiglec-7 is shed from the cellular surface on NK cells since high levels of HIV-1 replication induce an early decrement of Siglec-7 specifically on this population and not on in monocytes/macrophages and CD8^pos^ T cells, which are the other two immune cells constitutively expressing this type-I lectin (10, 52, 56). Even though the mechanisms, the kinetic, and the pathogenic relevance of the sSiglec-7-mediated infection of CD4^pos^ T cells are still unknown, it is conceivable to hypothesize that this c-lectin-type molecule could serve as a highly efficient and T-trophic carrier of the virus in the bloodstream and tissue sites (Figure 1).

## Siglec-7 and Inflammation in HIV-1 Infection

Since from the very beginning of HIV-1 pandemic, it became clear that the occurrence of a persistent inflammation is a hallmark of the disease together with the establishment of viral reservoirs (108, 109). Chronic inflammation also leads to a long-term activation of immune system even in the presence of ART and low levels of viremia (110). Several HIV-1-mediated mechanisms have been reported to induce an aberrant state of long-lasting inflammation, including the pathologic translocation of commensal micro-flora from the mucosal interfaces to both lamina propria and mesenteric lymph nodes in response to the massive depletion of mucosal CD4^pos^ T occurring during the acute phases of the infection (111). Macrophages, other then constitutively expressing several members of the Siglec family, also represent the sentinels of the host innate immune system against pathogens at mucosal interface (5, 112). In the case of HIV-1 infection, the persistent engagement of inflammatory pathway in macrophages chronically challenged by the virus itself or by several opportunistic pathogens likely contributes to induce a chronic state of immune activation. Indeed, it has been reported that Siglec-7 on MDMs is able to bind different pathogens either expressing sialic acid residues (i.e., HIV-1, K1 strain of *Escherichia coli* and *Candida albicans)* or not expressing syalilated glycans (i.e., K12 strain of *E. coli* and *Zymosan* yeast particles) (17, 52). Interestingly and in contrast with the reported inhibitory function of Siglec-7 in regulating the homeostasis and/or the effector functions of T and NK cells (113–115), the engagement of this type-I lectin on monocytes induces the production of several pro-inflammatory cytokines such as IL-6, IL-1α, CCL4, IL-8, and TNF-α (17). As a matter of fact, although coupled with ITIMs in its cytoplasmc domain, Siglec-7 promotes activation of monocytes *via* the engagement of an unconventional activating signal transduction pathway of these inhibitory motifs. It is well-known that ITIMs usually induce the phosphorylation of Src family kinases that, in turn, recruit phosphotyrosine phosphatase (PTP) such as Src homology region 2 domain-containing phosphatase-1 (SHP-1) and SHP-2. These PTPs switches off cellular pathways by dephosphorylating tyrosine residues and also prevents the activation signals that originate from receptors coupled with ITAMs (116). Recently, the interpretation of this dichotomy in signal transduction has been revised due to the identification of new transmembrane receptors containing ITIMs. Indeed, several studies demonstrated that, in some circumstances and depending on the cellular context, ITIMs can propagate activation signals and ITAMs can mediate inhibition (117–124). One example is given by the dendritic-cell-associated C-type lectin 2, an ITIM bearing receptor, whose cross-linking with an anti-DCAL-2 monoclonal Ab (mAb) recruits a SHP-2 domain that, instead of negatively regulating cell signaling, is involved in activating the ERK pathway and in inducing the production of IL-10, TNF-α, IL-6, and CCL5 (117). This phenomenon is also occurring with Siglec-7 isoform selectively expressed on monocyte and bearing “activating” ITIMs inducing the phosphorylation of ERK. This latter well-known signaling pathway triggers various transcription factors modulating many genes involved in the immune activation (125). Therefore, the encounter of different pathogens by Siglec-7 expressed on monocytes and macrophages likely provide an additional mechanism contributing to the appearance of persistent inflammation during the course of HIV infection.

Besides inducing the production of pro-inflammatory cytokines and chemokines in monocytes and macrophages, the cross-linking of Siglec-7 also induces an up-modulation of ICAM-1 and CD49e. These two adhesion molecules are known to play a relevant role in the trans-endothelial migration of leukocytes during inflammatory responses following their binding with either the β2 integrin lymphocyte function-associated antigen (LFA-1) or macrophage 1 antigen expressed on endothelial cells. Moreover, the interaction between ICAM-1 and LFA-1 is able to deliver a co-stimulatory signal to T cells (126). Therefore, the engagement of Siglec-7 has also the potential to regulate the migration of other inflammatory cells toward tissue sites, where they deliver co-stimulatory signals. This is highly relevant at gut and cervix mucosal interfaces, as these two tissue sites highly enriched of macrophages represent the main gates of HIV-1 entrance (127, 128). The activation and the migration of monocytes to tissues can lead to cellular activation and plastic polarization toward pro-inflammatory M1 or anti-inflammatory M2 macrophages on the basis of different signals and stimuli delivered within the local microenvironment (112). Further investigations are required to better understand the impact of HIV-1 binding to Siglecs constitutively expressed on macrophages in the modulation of their plasticity toward a classic or alternative polarization, a process that is known to be highly affected and dysfunctional during the course of HIV-1 infection (85). This is highly relevant in the context of the synergic interplays by NK cells and macrophages aimed to induce and optimize the links between innate and adaptive immune responses. In particular, the NK cell-macrophage cross talk occurring through mechanisms requiring both cell-to-cell contacts and soluble mediators is key in the context of host–pathogen interactions (129–131) and during the course of viral infections (132). Indeed, it has been recently reported that M1 macrophages can prime the activation and the effector functions of NK cells that, in turn, are able to rescue M2 toward pro-inflammatory M1 polarization (133). This virtuous loop appears to be completed disrupted during the course of chronic and active HIV-1 infections that is characterized by a macrophage deactivation or M2 polarization and by the expansion of anergic CD56^neg^ NK cells also impaired in the priming of autologous DCs (73, 75, 85). The cellular and molecular mechanisms behind the appearance of markedly dysfunctional NK cells and macrophages are still unknown and not even the selective down-modulation of Siglec-7 on NK cells has been clarified. Another important pathogenic element to elucidate is why the chronic HIV-1 replication induces the polarization of M2 macrophages that are also more susceptible to the infection through the engagement of several surface receptors including Siglec-7 (52, 90, 99). Finally, we do no know why HIV-1 binding to Siglec-7 does not induce a the polarization of macrophage toward M1 with a consequent priming of autologous NK cell able to better function as antiviral effectors. The elucidation of all these points will help us to better understand the basis of innate immune dysfunctions during the course of HIV-1 infection.

## Siglecs as Possible Therapeutic Targets in HIV-1 Infection

Targeting Siglecs as novel immunotherapy approaches has been proposed soon after the identification of Siglec-3 (CD33) and Siglec-2 (CD22) as biological markers of myeloid leukemias and B cell lymphomas, respectively (4). Indeed, the anti-Siglec-3 mAb Gemtuzumab ozogamicin is approved for treatment of acute myeloid leukemia, while Epratuzumab, the mAb targeting Siglec-2, is currently in clinical trials for treatment of acute lymphoblastic leukemia, follicular non-Hodgkins lymphoma, and autoimmune diseases (134–136). The identification of novel members within the Siglec family and the recent new insights disclosing the pathogenic relevance of this type-I lectins in several model of human diseases shed new light for developing innovative therapeutic approaches targeting the immune-modulatory functions of these transmembrane receptors. In particular, the existence of peculiar functions exerted by several Siglecs in distinct immune cell populations make these lectin-type molecules suitable candidates for therapies modulating host–pathogen interactions and cellular signal transduction.

In the context of HIV-1 infection, other then using Siglec-1 and Siglec-7 as possible clinical biomarkers, we can take advantage of their ability of binding HIV-1 envelope and enhance/induce the susceptibility to infections of both conventional and unconventional cell targets (52–55). Indeed, the use of pharmaceutical compounds or mAbs blocking the interactions of HIV-1 with either Siglec-1 or Siglec-7 can potentially limit HIV-1 dissemination in several cellular compartments (i.e., monocytes/macrophages, DCs, and CD4^pos^ T cells). This approach is particularly relevant during acute infection in order to damp the ability of HIV-1 to infect immune cells, to slow down the CD4^pos^ T cell depletion especially at mucosal interfaces and to put a break to the early establishment of immune activation and viral reservoirs. Furthermore, inhibiting HIV-1 uptake by Siglec-1 would also limit the infection of circulating monocytes that upregulate the surface expression of this type-I lectin in the acute phases of the infection (78, 79). Another possible therapeutic target is sSiglec-7 that is increased in the sera of AIDS patients and enhances the degree of infection of CD4^pos^ T cells (52). The elucidation of the mechanism(s) inducing the down-modulation of Siglec-7 on NK cell surface and inducing the shedding of its soluble form in AIDS patients’ sera will certainly make it possible to develop strategies that will limit the ability of sSiglec-7 to spread the infection due to its capacity to carrier HIV-1 in periphery and enhance the susceptibility to infection of Siglec-7^neg^/CD4^pos^ T cell targets.

In the context of developing a vaccine strategy, it has been also reported that Siglec-1 can be targeted to improve antigen presentation to T cells on the basis of its effective and rapid endocytic activity following interaction with pathogens (137, 138). Further investigations are needed to assess whether such approach can be used for HIV-1. Finally, it is also possible to take advantage of the immune-modulatory potential of Siglecs by employing their natural ligands. In this regard, it has been recently reported that high-affinity synthetized glycans targeting Siglec-2 expressed on B cells compete in *cis* with the natural ligands and induce endocytosis and killing of B cells (139). A similar approach could be used to inhibit the interactions between Siglecs and HIV-1. Particularly, attractive from a pharmaceutical perspective would be the use of lyposomal nanoparticles bearing Siglec glycan ligands (140).

## Concluding Remarks

Among the several mechanisms that HIV-1 has developed to evade host immune defenses there is the heavily conjugation on Gp120 of viral envelope with sialic acids residues that bind Siglec-1 and -7 mostly expressed on NK cells, macrophages, and DCs. These interactions are relevant for HIV-1 survival, serve as molecular mimicry for host immune responses, avoid immune attack, and facilitate viral entry. Indeed, the binding of HIV-1 to these type-I lectins induce and/or enhance the infections of target cells is associated with inflammatory pathways and correlated with disease progression (Figure 1). These novel insights disclosing the key roles played by Siglecs in the pathogenesis of HIV-1 infection open new avenues also for clinical perspectives. Indeed, this transmembrane type-I lectin could be either used as biological marker of disease or therapeutically targeted to limit viral dissemination and the establishment of viral reservoirs. Siglec-7 and Siglec-1 dysfunctions in HIV-1 infection are also highly relevant to advance our knowledge in regard to the physiology and physiopathology of NK cells either alone or in the context of their interplays with both monocytes/macrophages and DCs.

## Author Contributions

JM and DM wrote the manuscript and compiled the citations. CV and EZ contributed to write the manuscript and drew the figures.

## Conflict of Interest Statement

The authors declare that the research was conducted in the absence of any commercial or financial relationships that could be construed as a potential conflict of interest.
